# Clustering of smoking, alcohol drinking and cannabis use in adolescents in a rapidly developing country

**DOI:** 10.1186/1471-2458-6-169

**Published:** 2006-06-27

**Authors:** David Faeh, Bharathi Viswanathan, Arnaud Chiolero, Wick Warren, Pascal Bovet

**Affiliations:** 1 University Institute for Social and Preventive Medicine, Lausanne, Switzerland; 2 Ministry of Health and Social Services, Victoria, Republic of Seychelles; 3 Office on Smoking and Health, National Centre for Chronic Disease Prevention and Health Promotion, Centers for Disease Control and Prevention, Atlanta, USA

## Abstract

**Background:**

Smoking, alcohol drinking and cannabis use ("risk behaviors") are often initiated at a young age but few epidemiological studies have assessed their joined prevalence in children in developing countries. This study aims at examining the joint prevalence of these behaviors in adolescents in the Seychelles, a rapidly developing country in the Indian Ocean.

**Methods:**

Cross-sectional survey in a representative sample of secondary school students using an anonymous self-administered questionnaire (Global Youth Tobacco Survey). The questionnaire was completed by 1,321 (92%) of 1,442 eligible students aged 11 to 17 years. Main variables of interest included smoking cigarettes on ≥1 day in the past 30 days; drinking any alcohol beverage on ≥1 day in the past 30 days and using cannabis at least once in the past 12 months.

**Results:**

In boys and girls, respectively, prevalence (95% CI) was 30% (26–34)/21% (18–25) for smoking, 49% (45–54)/48% (43–52) for drinking, and 17% (15–20)/8% (6–10) for cannabis use. The prevalence of all these behaviors increased with age. Smokers were two times more likely than non-smokers to drink and nine times more likely to use cannabis. Drinkers were three times more likely than non-drinkers to smoke or to use cannabis. Comparison of observed versus expected frequencies of combination categories demonstrated clustering of these risk behaviors in students (P < 0.001).

**Conclusion:**

Smoking, drinking and cannabis use were common and clustered among adolescents of a rapidly developing country. These findings stress the need for early and integrated prevention programs.

## Background

In addition to the increased risk of chronic diseases at an older age, smoking, drinking and use of illegal substances in adolescents are associated with more immediate health hazards such as depression, interpersonal violence, motor vehicle crashes and drowning, risky sexual behaviors, and suicidal behavior [1-3]. Furthermore, behaviors initiated during adolescence tend to track into adulthood [4]. Early experience with smoking and drinking increases the risk of subsequent tobacco [5] and alcohol [6] dependences. In addition, cross-sectional [7-9] and longitudinal [10-12] studies in western countries have shown that these behaviors tended to cluster in adolescence and perhaps even at an earlier age [13]. Also of importance, these behaviors increase the likelihood to adopt other risk behaviors at a later age, such as multiple substance use, violence and delinquency [14,15].

The Republic of Seychelles comprises over 115 islands lying in the Indian Ocean, approximately 1,000 km east to Kenya and the country is part of the African region. Almost all of the population lives on three main islands and 90% reside on the largest one, Mahé. The large majority is of African descent. The country has experienced rapid socio-economic development due to booming tourism and fishing industries. The gross domestic product per capita has increased, in real terms, from US$ 2,927 in 1980 to US$ 5,239 in 2004.

Surveys among adults have shown high prevalence of both smoking and drinking in men but not in women [16-18]. In 1994, 51% of men and 6% of women reported drinking at least once per week [17] while 31% of men and 4% of women reported smoking daily in 2004 [18]. Until 2004, drugs such as heroin or cocaine were virtually unseen in the country, which was reflected by only very few police cases or hospital admissions related to illegal substance abuse.

In Seychelles, the law prohibits the sale of alcohol and cigarettes to children aged less than 18 years. Use and possession of cannabis and other illegal drugs is prohibited and liable to severe penalties. Both alcohol and cigarettes are heavily taxed and expensive. Advertising for cigarettes is banned while advertising for alcohol beverages is limited. Use of tobacco or alcohol in the premises of all schools is prohibited and severely sanctioned by school policies. Over the past decade, the ministry of health and some other organizations have been conducting high-profile awareness campaigns related to smoking, illegal drugs and responsible drinking. However, alcohol drinking is common at the occasion of social events and there has been a long standing social tolerance, at least among men [17].

The prevalence, age of onset and clustering of smoking, drinking and substance use in adolescents has been well described in western countries but few data on the joint prevalence of these behaviors are available from developing countries. In this study, we examined the prevalence and clustering patterns of smoking, drinking and cannabis use among a representative sample of school students aged 11–17 years of the Seychelles, a middle-income country experiencing rapid epidemiological transition.

## Methods

This study is part of the Global Youth Tobacco Survey (GYTS), an international school-based survey of tobacco use that focuses on adolescents aged 13–15. The survey, which is sponsored by the World Health Organization (WHO) and the Centers for Disease Control (CDC), has been conducted once or several times in more than a hundred countries worldwide [19-22].

The GYTS is intended to be performed in students aged 13–15 years. In Seychelles this includes the four secondary grades S1–S4 (school is compulsory through the S4 level). There are 12 secondary schools (10 public, 2 private) on the three main islands that teach grades S1–S4. The total enrolment of Grades S1–S4 for these 12 schools was 6,161. A two-stage cluster sample design was used to produce a representative sample of all students in grades S1–S4 from all public and private schools in Seychelles. The first-stage sampling frame consisted of all schools containing the grades S1, S2, S3, and S4. Schools were selected with probability proportional to school enrolment size. The second-stage sampling frame consisted of an equal-probability sampling (with a random start) of all S1–S4 classes from the selected schools. Sample size estimation showed that 1,224 completed interviews were needed from an enrolment of 6,161 for a ± 5% margin of error.

Eight percent of students did not participate in the study, because they were absent on the day the study took place for benign reasons (e.g. illness) or because they were suspended for disciplinary reasons (i.e. major misbehaviors) [23].

The questionnaire was anonymous, self-administered, and written in English (English is the main language used at school). The questionnaire included 56 core questions on tobacco and other variables (for example age, sex) used in all GYTS worldwide. Fifteen additional questions were included in order to assess alcohol drinking and the use of illegal drugs [see Additional file 1]. Most students could complete the questionnaire within 35–45 minutes. The survey took place at the same time in all selected classes. The students and parents were not informed prior to the survey in view of the non-sensitive nature of the survey, the absence of invasive investigations or physical measurements, the allowance for declining participation given to all children, and the anonymous nature of the questionnaire ensuring confidentiality of all answers by all students. The research committee of the Ministry of Health and the Ministry of Education approved the study including the questionnaire and the fact that informed consent by parents was not necessary.

Some questions of particular relevance for this study read as follows: "During the past 30 days, on how many days did you smoke cigarettes?". Consistent with the GYTS methodology, "smoking in the past month" was defined as smoking on one day per month or more. "Have you ever tried cigarette smoking, even one or two puffs?". "Ever smoking" was defined as having ever smoked a cigarette, even a puff, at least once. "During the past 30 days, on how many days did you drink alcohol?". "Drinking in the past month" was defined as any alcohol consumption on at least one day during the past 30 days. "Have you ever had so much alcohol that you were drunk?". "Ever drunkenness" was defined as having ever been drunk at least once. "Did you ever take a joint, marijuana, or hashish in your life?". "Ever cannabis" was defined as having ever used cannabis at least once. "During the past 12 months, how many times did you take a joint, marijuana, or hashish?". "Cannabis in the past year" was defined as having smoked cannabis at least once during the past 12 months [see Additional file 1].

Overall prevalence estimates and 95% confidence intervals were weighted consistent with the survey design. We selected age categories (11–13, 14 and 15–17) so that numbers of students were balanced across all categories in order to maximize the power of statistical analyses. Differences in prevalence estimates were tested using the chi-square test. To analyze clustering patterns, we calculated the expected frequencies of the 4 different possible combinations of the three risk factors (i.e. 0, 1, 2, or 3) given the observed prevalence of each risk factor and assuming independency in associations. Difference between observed versus expected prevalences of combinations was tested with a goodness-of-fit chi square with 3 degrees of freedom. Analyses were conducted with Stata 8.2. P-values less than 0.05 were considered significant.

## Results

All schools agreed to participate (100% school response rate) and 1,321 (92%) of 1,442 eligible students aged 11 to 17 years completed the questionnaire. Forty-two percent of all participants were aged 11–13, 28% aged 14, and 30% aged 15–17. The proportions of respondents did not differ significantly by age and age category. The answers not completed in the questionnaire were coded as missing values and their proportions varied between 0.9% and 5.6% depending on the questions.

Table 1 presents the proportions of boys and girls reporting risk behaviors. The prevalence of risk behaviors was markedly higher in the older than younger age categories. This suggests an important uptake of these risk behaviors over the considered age categories. The prevalence of risk behaviors was higher in boys than girls. However, at age 15–17, the difference between boys and girls was only minimal for monthly smoking and for monthly drinking, suggesting convergence in the prevalence in late adolescence. The prevalence of cannabis use in the past year was larger in boys than in girls in all age categories.

Table 2 shows the proportions of combinations of risk behaviors by sex and age categories. The proportions of students reporting none of the three risk behaviors tended to be lower in boys than in girls and in younger than older students (e.g. 41% at age 11–13 and 24% at age 15–17 among boys, respectively 50% and 35% among girls of same age categories). Inversely, the proportions of students reporting the three risk behaviors tended to be higher in boys than in girls and in the older than younger age categories (e.g. 7% at age 11–13 and 14% at age 15–17 among boys, respectively 3% and 8% among girls in the same age categories).

**The figure **shows the prevalence of combinations of 0, 1, 2 and 3 risk behaviors among all students. The prevalence of expected and observed combinations categories differed significantly (P < 0.001), which demonstrates a clustering pattern of the considered risk behaviors. Observed prevalences were higher than expected prevalences in the extreme combination categories (respectively 0 and 3 risk behaviors) while the reverse was found in the intermediate combination categories (respectively 1 and 2 risk behaviors). Compared to the expected prevalence, the observed prevalence was +33% (45% vs. 34%), -30% (34% vs. 48%), -15% (14% vs. 17%) and +380% (7% vs. 2%) in the combination categories of 0, 1, 2 and 3 risk behaviors, respectively. Results did not differ significantly by sex or age (data not shown).

Table 3 shows the joint prevalence of risk behaviors among smokers (n = 294), drinkers (n = 613) and cannabis users (n = 154). Compared to non-smokers, smokers were two times more likely to drink (76% vs. 38%), and more than nine times more likely to use cannabis (35% vs. 4%). Compared to non-drinkers, drinkers were three times more likely to smoke (39% vs. 11%) or to use cannabis (20% vs. 6%). Compared to non-users, cannabis users were four time more likely to smoke (75% vs. 18%) and two times more likely to drink (77% vs. 44%). Among drinkers, there were twice as many smokers as cannabis users (39% vs. 20%) and cannabis use was more common among smokers than among drinkers (35% vs. 20%). The 95% confidence intervals of prevalence estimates by "yes" and "no" status fell largely apart of each other in all situations shown in the table, which translates into significant differences in all instances.

Table 4 presents selected recently published estimates of the gender-specific prevalence of smoking, drinking and cannabis use among youth the United States [24], England [25], Switzerland [26], and South Africa [20,27], in addition to our findings in Seychelles. We did not find publications on the joint prevalence of the considered behaviors in representative samples in African countries except for one in South Africa. Although the mean age of the study populations and the criteria used to define risk behaviors differ across countries, – and the figures must therefore be interpreted with great caution -, the table may be useful to set the prevalence of risk behaviors among Seychelles youth in an international context. The data suggest that the prevalence and gender patterns for smoking and drinking are not largely different in Seychelles as compared to the considered western countries. However fewer youths seemed to use cannabis in the former than in the latter.

## Discussion

We found high prevalences of smoking, drinking and cannabis use among adolescents in the Seychelles. These risk behaviors tended to cluster, particularly smoking and drinking and smoking and cannabis.

Comparisons between countries must be interpreted cautiously in view of different methodology used across countries. However, the prevalence of smoking seemed higher in Seychelles than in several low-income developing countries in the African region [20], but similar as compared to western countries such as Switzerland [28], the US [24] or the UK [25]. Higher prevalence in Seychelles as compared to Africa may reflect a larger purchasing power of youth in Seychelles than in many other African countries [29]. While smoking is much less common in female than in male adults in Seychelles (respectively 4% and 31% in 2004 [30]), the much smaller difference by gender among youth may predict a marked increase in the prevalence of smoking in future generations of adult women [17,31]. A lack of gender differences in adolescent smoking has been consistently found in developed and developing countries (Table 4) [24,25,28,32].

Drinking in adolescents was as frequent in Seychelles as in South Africa [27] or in selected western countries [24,25,28]. The lack of a gender difference in drinking prevalence in Seychellois adolescents, consistent with findings in industrialized countries (Table 4) [24,25,28,32], may announce a convergence by gender among younger cohorts, in contrast to the currently much higher prevalence of drinking in male than female adults [16-18].

Fewer adolescents in Seychelles reported cannabis use during the past year compared to youths in South Africa or in some western countries (Table 4). A higher prevalence in boys than in girls in Seychelles contrasts with the lack of a gender difference observed in selected other countries. The situation may be consistent with the indirect evidence of a low use of other illegal drugs in Seychelles at the time of the survey.

We found that smoking, drinking and cannabis use tended to cluster. The association was particularly strong between smoking and cannabis use, which is consistent with a clustering pattern often found in developed countries [1,33]. The cross-sectional nature of our data precludes concluding on the direction of these associations (whether a certain behavior precedes or follows another behavior). Studies investigating temporal relationships of substance use suggest that smoking often precedes alcohol drinking and cannabis use and that smoking may represent a "gateway" to other substance use [14,15,34-37]. On the other hand, a "problem behaviors theory" postulates that there is no clear temporal sequence in the use of different substances. Along this theory, adoption of risk behaviors such as smoking, drinking, cannabis use and the use of other drugs is the consequence of a single underlying characteristic [38], e.g. an affinity to sensation-seeking and risky behaviors [39]. The associations between risk behaviors may also reflect social circumstances such as use of substances by peers and a perception by children that use of substances is encouraged among adults [40]. Pharmacologic factors may also play a role: tobacco, alcohol, cannabis and other drugs impact on several common neurotransmitters [41]. Moreover, early onset of smoking, drinking or cannabis use has been associated with higher nicotine addiction [4], higher alcohol dependence [6] and increased problem behaviors [42] during later adolescence, which may underlie other mechanisms for the association of these behaviors.

Mirroring a higher than expected prevalence of adolescents with all three behaviors, we also found a larger than expected proportion of adolescents indulging in none of the three risk behaviors. One could speculate that abstainers might include adolescents who socialize less and are therefore less exposed to peer pressure from experimenters or who are submitted to more parental or other social or cultural forms of control. Alternatively, abstainers might have developed particularly strong resilience skills, in relation to personal characteristics or to an enabling familial or social milieu.

Our study has some limitations. The sample size of the survey limits the precision of the prevalence estimates in some categories with subsequent large confidence intervals (e.g. cannabis use among girls). The size of the samples if further limited by missing data in some students (typically up to 5%), a factor that cannot be avoided from self-reported questionnaires. There are also several sources of bias in prevalence estimates. First, students could exaggerate or underestimate their answers in a systematic manner. Although the extent of this potential bias cannot be determined, questions on tobacco were shown to have good test-retest reliability in a another study [20]. Second, the prevalence of risk behaviors may be underestimated since non-participants typically indulge in more detrimental behaviors as compared to participants, as we have also shown on this sample of students [23]. On the other hand, strong points of the study include the large participation, the population-based design, and the anonymous nature of the data collected.

Our findings have several public health implications. First, the early onset of the considered behaviors emphasizes the importance of implementing school-based surveillance systems for guiding and evaluating policy and prevention programs.

Second, given the much lower prevalence of smoking and drinking in women compared to men in Seychelles (as found in many other developing countries), the only small gender difference found in boys and girls may predict a convergence by sex of these behaviors over the next decades. However, further appropriately powered studies (i.e. studies with larger sample sizes and extended age categories) should examine trends and significance of such gender patterns over age in adolescence before conclusions can be drawn. These issues are important as they underlie specific target groups for prevention.

Third, given tracking into adulthood [4,6], the fairly high prevalence of the considered risk behaviors in adolescents emphasizes the need to strengthen school-based programs and policies aimed at promoting healthy behaviors as a main strategy to tackle non-communicable diseases in the general population. It has been shown that delaying smoking onset until after the age of 13 can reduce the prevalence of adult smoking [43] and that delaying drinking onset after the age of 14 can reduce the risk of alcohol dependence [6]. More generally, it is believed that adoption of healthy behaviors early in life has the potential to reduce CVD in adulthood [44]. Furthermore, programs to prevent substance use should therefore not be restricted to adolescents only but also extend to pupils in elementary school.

Fourth, the trend towards clustering of smoking, drinking and use of cannabis, as found in this report and in other studies, emphasizes the need to address these behaviors within comprehensive and integrated programs [43]. Similarly, comprehensive benefits can be expected from various policy measures: high cigarette prices can reduce the prevalence of both drinking and cannabis use in adolescents [45].

## Conclusion

We found that smoking, drinking and cannabis use are common among adolescents of a rapidly developing country and that these risk behaviors are adopted at an early age and tend to cluster. These findings stress the need to initiate prevention interventions at an early age and using integrated approaches.

## Competing interests

The author(s) declare that they have no competing interests.

## Abbreviations

GYTS: Global Youth Tobacco Survey

WHO: World Health Organization

CDC: Centers for Disease Control

## Authors' contributions

DF carried out literature research, statistical analysis, assisted in interpretation of the data, and was the main writer of the manuscript. BV participated in the study design and collected the data. AC and WW assisted in statistical analysis and reviewed the manuscript. PB designed the study, carried out analysis and interpretation of the data, and wrote partially the report.

## Pre-publication history

The pre-publication history for this paper can be accessed here:



## Supplementary Material

Additional file 1 Final-GYTS-questionnaire(17sep02). Global Youth Tobacco Survey (GYTS) questionnaire with additional questions on alcohol consumption and use of illegal substances. Click here for file

## Figures and Tables

**Figure 1. Prevalence of expected and observed combinations of risk behaviors (smoking, drinking and cannabis use) in students aged 11–17:**
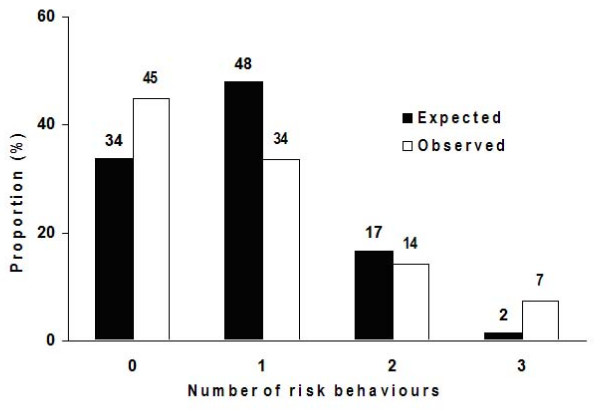
Prevalence of expected and observed combinations of risk behaviors (smoking, drinking and cannabis use) in students aged 11–17

**Table 1:** Prevalence (percent with their 95% confidence intervals) of students reporting risk behaviors, by sex and age

	Age	11–13 years		14 years		15–17 years		Total	
					
		%	95% CI	%	95% CI	%	95% CI	%	95% CI
Boys	n	257		160		203		620	
	Ever smoking	46	38 - 54	53	45 - 61	67	60 - 73	54	50 - 59
	Smoking in the past month	27	20 - 35	24	18 - 31	38	30 - 45	30	26 - 34
	Drinking in the past month	45	38 - 59	40	35 - 46	63	56 - 69	49	45 - 54
	Ever drunkenness	40	33 - 47	44	35 - 53	58	50 - 66	47	42 - 61
	Ever cannabis	15	10 - 22	15	11 - 21	29	23 - 37	20	17 - 22
	Cannabis in the past year	15	10 - 19	12	8 - 18	26	20 - 33	17	15 - 20
Girls	n	274		193		187		654	
	Ever smoking	30	24 - 36	44	36 - 52	56	50 - 62	41	38 - 45
	Smoking in the past month	15	10 - 22	23	18 - 29	28	22 - 35	21	18 - 25
	Drinking in the past month	41	34 - 48	47	39 - 55	58	49 - 65	48	43 - 52
	Ever drunkenness	35	29 - 42	44	37 - 51	52	45 - 60	43	39 - 47
	Ever cannabis	6	4 - 9	6	3 - 12	16	12 - 21	9	7 - 11
	Cannabis in the past year	6	4 - 10	5	2 - 11	12	9 - 18	8	6 - 10

**Table 2:** Prevalence (percent with their 95% confidence intervals) of students reporting various combinations of smoking in the past month (S), drinking in the past month (D) and cannabis use in the past year (C) by sex and age. Y: Yes, N: No

	Age				11–13 years		14 years		15–17 years	
							
	S	D	C	n	%	95% CI	%	95% CI	%	95% CI
Boys	N	N	N	226	41	34–49	44	36–52	24	18–30
	Y	N	N	31	5	3–9	5	2–10	5	3–10
	N	Y	N	135	20	15–27	18	13–24	27	20–35
	N	N	Y	14	2	1–4	4	2–9	2	1–6
	Y	Y	N	58	9	6–13	10	7–15	9	6–14
	N	Y	Y	12	2	1–4	1	0–5	3	1–7
	Y	N	Y	11	1	0–3	1	0–5	4	2–7
	Y	Y	Y	54	7	4–11	5	2–11	14	10–20
Girls	N	N	N	286	50	43–57	43	36–50	35	27–43
	Y	N	N	20	3	1–7	3	1–7	4	2–8
	N	Y	N	180	25	20–32	28	21–35	31	25–38
	N	N	Y	19	4	2–8	4	2–7	1	0–4
	Y	Y	N	72	7	4–13	14	10–20	13	9–18
	N	Y	Y	5	1	0–3	1	0–4	1	0–4
	Y	N	Y	4	<1	0–3	1	0–4	1	0–4
	Y	Y	Y	28	3	1–6	3	1–8	8	5–13

**Table 3:** Joint prevalence of risk behaviors by sex

		Smokers			Drinkers			Cannabis users		
				
	n	Yes (%)	No (%)	Ratio	Yes (%)	No (%)	Ratio	Yes (%)	No (%)	Ratio
Smoking in the past month										
Boys	164	100	-	-	43	16	2.7	76	20	3.8
Girls	130	100	-	-	35	8	4.7	75	17	4.5
Total	294	100	-	-	39	11	3.4	75	18	4.1
Drinking in the past month										
Boys	304	71	39	1.8	100	-	-	76	44	1.7
Girls	309	81	39	2.1	100	-	-	77	45	1.7
Total	613	76	38	2.0	100	-	-	77	44	1.7
Cannabis in the past year										
Boys	105	42	6	7.6	26	8	3.2	100	-	-
Girls	49	26	2	11.4	12	3	3.7	100	-	-
Total	154	35	4	9.3	20	6	3.5	100	-	-

Prevalence of risk behaviors by "Yes" vs. "No" status differed significantly (p < 0.05) in all instances. Smokers: smoking in the past month; Drinkers: drinking in the past month; Cannabis users: Cannabis use in the past year.

**Table 4:** Prevalence of risk behaviors in adolescents in Seychelles and in selected countries

	Proportion (%)			
	
	Age (y)	Boys	Girls	Total
**Ever smoking**				
Seychelles*	11–17	54	41	48
South Africa (a)	13–15	-	-	44
England^£^	11–15	37	41	39
USA^$^	14–18	59	58	58
Switzerland^&^	15	64	64	64
**At least 1 cigarette in the past month**				
Seychelles	11–17	30	21	25
South Africa (a)	13–15	-	-	18
USA	14–18	30	35	28
England^1^	11–15	11	17	14
Switzerland	15	22	21	21
**At least 1 drink in the past month**				
Seychelles	11–17	49	48	48
South Africa (b)	16–17	50	32	-
USA	14–18	44	46	45
England	11–15	37	39	38
Switzerland	15	77	74	75
**Ever drunkenness**				
Seychelles	11–17	47	43	45
USA^2^	14–18	29	28	28
England^3^	11–15	42	50	46
Switzerland	15	64	53	58
**Ever used cannabis**				
Seychelles	11–17	20	9	14
South Africa (b)	16–17	32	13	-
USA	14–18	43	38	40
Switzerland	15	44	36	40
**Cannabis use in the past year**				
Seychelles	11–17	17	8	12
South Africa (b)	16–17	22	9	-
USA	14–18	25	19	22
England	11–15	12	10	11
Switzerland	15	35	28	31

*Seychelles: n = 1,321, 2002.

^£^England: n = 9,572, 2004, reference [25]; ^$^USA: n = 15,214, 2003, reference [24].

^&^Switzerland: n = 2,613, reference [26]

South Africa (a): n = 2,579, 2002, reference [20]; South Africa (b): Cape Town, n = 2,732, 1997–2001, reference [27].

^1 ^Regular occasional smoking.

^2 ^At least 5 drinks in a row.

^3 ^Among students who drank in the past week.
