# Supplementary material for: Clustering of smoking, alcohol drinking and cannabis use in adolescents in a rapidly developing country
Source: BMC Public Health. 2006 Jun 27;6:169. doi: 10.1186/1471-2458-6-169 (PMC1564395; doi:10.1186/1471-2458-6-169)
Supplement: Additional file 1 — Final-GYTS-questionnaire(17sep02). Global Youth Tobacco Survey (GYTS) questionnaire with additional questions on alcohol consumption and use of illegal substances. [file 1471-2458-6-169-S1.pdf]

CORE QUESTIONS

GLOBAL YOUTH TOBACCO SURVEY (GYTS)

2002

Dear Student,

By answering to this questionnaire, you will help us to understand how students of your age live, particularly in relation to lifestyles and health. These very same questions have been recently asked to thousands of students in several countries worldwide.

Please, do <u>not</u> write your name on this questionnaire. Hence, nobody will be able to identify who has completed this particular form. Please, answer honestly to all the questions.

Please answer to the questions in the order they appear. This is not a test; therefore there are no 'right' or 'wrong' answers.

On the answer sheet, locate the circle that corresponds to your answer and fill it in completely with the pencil that was provided to you.

Correctly fill in the bubbles:

☺ Like this: ● ☹ Not: ○ or x

Example:

Questionnaire	
24. Do you believe that fish live in water?<br>a. Definitely yes<br>b. Probably yes<br>c. Probably not<br>d. Definitely not	24. ● B C D E F G H

Thank you a lot for your collaboration.

THE NEXT 14 QUESTIONS ASK ABOUT YOUR USE OF TOBACCO.

1. Have you ever tried cigarette smoking, even one or two puffs?

a. Yes
b. No

2. How old were you when you first tried a cigarette?

a. I have never smoked cigarettes
b. 7 years old or younger
c. 8 or 9 years old
d. 10 or 11 years old
e. 12 or 13 years old
f. 14 or 15 years old
g. 16 years old or older

3. During the past 30 days (one month), on how many days did you smoke cigarettes?

a. 0 days
b. 1 or 2 days
c. 3 to 5 days
d. 6 to 9 days
e. 10 to 19 days
f. 20 to 29 days
g. All 30 days

4. During the past 30 days (one month), on the days you smoked, how many cigarettes did you usually smoke?

a. I did not smoke cigarettes during the past 30 days (one month)
b. Less than 1 cigarette per day
c. 1 cigarette per day
d. 2 to 5 cigarettes per day
e. 6 to 10 cigarettes per day
f. 11 to 20 cigarettes per day
g. More than 20 cigarettes per day

5. During the past 30 days (one month), how did you get your own cigarettes most often? (SELECT ONLY ONE RESPONSE)

a. I did not smoke cigarettes during the past 30 days (one month)
b. I bought them in a store or a shop
c. I gave someone else money to buy them for me
d. I borrowed them from someone else
e. I stole them
f. An older person gave them to me
g. I got them some other way

6. During the past 30 days (one month), what brand of cigarettes did you smoke most often? (SELECT ONLY ONE RESPONSE)

a. I did not smoke cigarettes during the past 30 days
b. Mixture of various brands
c. Sword Fish
d. Mahe King
e. Benson & Hedges
f. 555
g. Marlboro
h. Other

7. During the past 30 days (one month), did you buy cigarettes by yourself for your own use?

a. I did not buy cigarettes during the past 30 days
b. Generally I buy 1 cigarette at a time
c. Generally I buy 2-3 cigarettes at a time
d. Generally I buy 4-5 cigarettes at a time
e. Generally I buy a pack of 10 cigarettes at a time
f. Generally I buy a pack of 20 cigarettes at a time

8. How much do you usually pay for a pack of 20 cigarettes that you smoke?

a. I don’t smoke cigarettes
b. I only buy cigarettes by units (1 or a few cigarettes at a time)
c. SR. 15 to 19 for 1 pack of 20 cigarettes d. SR. 20 to 24 for 1 pack of 20 cigarettese. SR. 25 to 29 for 1 pack of 20 cigarettes
f. SR. 30 to 34 for 1 pack of 20 cigarettes
g. SR. 35 to 49 for 1 pack of 20 cigarettes h. SR. 50 and more for 1 pack of 20 cigarettes

9. During the past 30 days (one month) how much did you spend on cigarettes?

a. I don’t smoke cigarettes
b. I smoke cigarettes but I don’t buy my cigarettes
c. Less than SR. 10
d. SR 11 to 20
e. SR 21 to 40
f. SR 41 to 60
g. SR 61 to 100
h. More than SR 100

10. In a usual month (30 days) how much pocket money (or income or allowance) do you get?

a. I don’t receive any pocket money (or income or allowance)
b. Less than SR. 5
c. SR 5 to 25
d. SR 26 to 50
e. SR 51 to 100
f. SR 100 to 150
g. More than SR 150

11. During the past 30 days (one month), did any shopkeeper ever refuse to sell you cigarettes because of your age?

a. I did not try to buy cigarettes during the past 30 days
b. I tried to buy cigarettes at a shop but the shopkeeper refused to sell me cigarettes because of my age
c. I tried to buy cigarettes at a shop and the shopkeeper sold it to me

12. During the past 30 days (one month), have you ever used any form of tobacco products other than cigarettes (e.g. chewing tobacco, snuff, dip, cigars, cigarillos, little cigars, pipe)?

a. Yes
b. No

13. Where do you smoke most often? (SELECT ONLY ONE RESPONSE)

a. I have never smoked cigarettes
b. At home
c. At school inside premises
d. At school outside premises
e. At friends’ placess
f. At social events (e.g. discos, fancy fairs)
g. In public places (e.g. in town, , streets, beach, near shops)
. h. Other

14. Do you ever smoke a cigarette or feel like smoking a cigarette first thing in the morning?

a. I have never smoked cigarettes
b. I no longer smoke cigarettes
c. No, I don’t smoke or feel like smoking a cigarette first thing in the morning
d. Yes, I sometimes smoke or feel like smoking a cigarette first thing in the morning
e. Yes, I always smoke or feel like smoking a cigarette first thing in the morning

THE NEXT 17 QUESTIONS ASK ABOUT YOUR KNOWLEDGE AND ATTITUDES TOWARD TOBACCO.

15. Do your parents (or steparents/guardians who stay at your home) smoke?

a. None
b. Both
c. Father or stepfather (boper) only
d. Mother or stepmother (manman swanye) only
e. I don’t know

16. If one of your best friends offered you a cigarette, would you smoke it?

a. Definitely not
b. Probably not
c. Probably yes
d. Definitely yes

17. Has anyone in your family discussed the harmful effects of smoking with you?

a. Yes
b. No

18. Do you think you will smoke a cigarette at any time during the next 12 months?

a. Definitely not
b. Probably not
c. Probably yes
d. Definitely yes

-19. Do you think you will be smoking cigarettes 5 years from now?

a. Definitely not
b. Probably not
c. Probably yes
d. Definitely yes

20.,Do you think it would be difficult to quit once someone has started smoking?

a. Definitely not
b. Probably not
c. Probably yes
d. Definitely yes

21. Do you think boys who smoke cigarettes have more or less friends?

a. More friends
b. Less friends
c. No difference from non-smokers

22. Do you think girls who smoke cigarettes have more or less friends?

a. More friends
b. Less friends
c. No difference from non-smokers

23 Does smoking cigarettes help people feel more or less comfortable at celebrations, parties, or in other social gatherings?

a. More comfortable
b. Less comfortable
c. No difference from non-smokers

24. Do you think smoking cigarettes makes boys look more or less attractive?

a. More attractive
b. Less attractive
c. No difference from non-smokers

25. Do you think smoking cigarettes makes girls look more or less attractive?

a. More attractive
b. Less attractive
c. No difference from non-smokers

26. Do you think that smoking cigarettes makes you gain or lose weight?

a. Gain weight
b. Lose weight
c. No difference

27. Do you think cigarette smoking is harmful to your health?

a. Definitely not
b. Probably not
c. Probably yes
d. Definitely yes

28. Do any of your closest friends smoke cigarettes?

a. None of them
b. Some of them
c. Most of them
d. All of them

29. When you see a man smoking what do you think of him? (SELECT ONLY ONE RESPONSE)

. a. He lacks confidence
b. He is stupid
c. He is aloser
d. He is successful
e. He is intelligent
f.He is a ‘real man’

30. When you see a woman smoking, what do you think of her? (SELECT ONLY ONE RESPONSE)

a. She lacks confidence
b. She is stupid
c. She is a loser
d. She is successful
e. She is intelligent
f. She is sophisticated

31. Do you think it is safe to smoke for only a year or two as long as you quit after that?

a. Definitely not
b. Probably not
c. Probably yes
d. Definitely yes

THE NEXT 4 QUESTIONS ASK ABOUT YOUR EXPOSURE TO OTHER PEOPLE'S SMOKING

32. Do you think the smoke from other people's cigarettes is harmful to you?

a. Definitely not
b. Probably not
c. Probably yes
d. Definitely yes

33. During the past 7 days, on how many days have people smoked in your home, in your presence?

a. 0
b. 1 to 2
c. 3 to 4
d. 5 to 6
e. 7

34. During the past 7 days, on how many days have people smoked in your presence, in places other than in your home?

a. 0
b. 1 to 2
c. 3 to 4
d. 5 to 6
e. 7

35. Are you in favor of banning smoking in enclosed public places (such as in restaurants, buses, schools, , gyms and sports arenas, discos)?

a. Yes in all enclosed places without exceptions
b. Yes in all enclosed places, but allowing for designated areas for smokers
c. No

THE NEXT 6 QUESTIONS ASK ABOUT YOUR ATTITUDES TOWARD STOPPING SMOKING

36. Do you want to stop smoking now?

a. I have never smoked cigarettes
b. I had smoked every day or occasionally in the past but I do not smoke now anymore
c. I smoke every day or occasionally and I would like to stop smoking
d. I smoke every day or occasionally and I do <u>not</u> want to stop smoking

37. During the past year, have you ever tried to stop smoking cigarettes?

a. I have never smoked cigarettes
b. I did not smoke during the past year
c. Yes
d. No

38. How long ago did you stop smoking?

a. I have never smoked cigarettes
b. I have not stopped smoking
c. I have stopped smoking 1-3 months ago
d. I have stopped smoking 4-11 months ago
e. I have stopped smoking 1 year ago
f. I have stopped smoking 2 years ago
g. I have stopped smoking 3 years ago or longer

39. What was the main reason you decided to stop smoking? (SELECT ONE RESPONSE ONLY)

a. I have never smoked cigarettes
b. I have not stopped smoking
c To improve my health
d. To save money
e. Because my family does not like it
f. Because my friends don't like it
g. Other

40. Do you think you would be able to stop smoking if you wanted to?

a. I have never smoked cigarettes
b. I have already stopped smoking cigarettes
c. Yes
d. No

41. Have you ever received help or advice to help you stop smoking? (SELECT ONLY ONE RESPONSE)

a. I have never smoked cigarettes
b. Yes, from a program or professional
c. Yes, from a friend
d. Yes, from a family member
e. Yes, from both programs or professionals and from friends or family members
f. No

42. Do your parents know that you smoke cigarettes?

a. I do not smoke cigarettes
b. Yes
c. No

THE NEXT 9 QUESTIONS ASK ABOUT YOUR KNOWLEDGE OF MEDIA MESSAGES ABOUT SMOKING

43. During the past 30 days (one month), how many <u>anti-</u>smoking media messages (e.g., on television, radio, billboards, posters, newspapers, magazines, movies) have you seen or heard?

a. A lot
b. A few
c. None

44. When you go to sports events, fairs, concerts, community events, or social gatherings, how often do you see <u>anti-</u>smoking messages?

a. I never go to sports events, fairs, concerts, community events, or social gatherings
b. A lot
c. Sometimes
d. Never

45. When you watch TV, videos, or movies, how often do you see actors smoking?

a. I never watch TV, videos, or movies
b. A lot
c. Sometimes
d. Never

46. Do you have something (t-shirt, pen, backpack, etc.) with a cigarette brand logo on it?

a. Yes
b. No

47. During the past 30 days (one month), when you watched sports events or other programs on TV how often did you see cigarette brand names (e.g. Marlboro) ?

a. I never watch TV
b. A lot
c. Sometimes
d. Never

48. During the past 30 days (one month), how many advertisements for cigarettes have you seen on billboards in Seychelles?

a. A lot
b. A few
c. None

49. During the past 30 days (one month), how many advertisements or promotions for cigarettes have you seen in newspapers or magazines in Seychelles?

a. A lot
b. A few
c. None

50. When you go to sports events, fairs, concerts, or community events in Seychelles how often do you see advertisements for cigarettes?

a. I never attend sports events, fairs, concerts, or community events
b. A lot
c. Sometimes
d. Never

51. Has someone working for cigarette companies ever offered you a free cigarette?

a. Yes
b. No

THE NEXT 4 QUESTIONS ASK ABOUT WHAT WAS DISCUSSED ABOUT SMOKING IN SCHOOL

52. During this school year (since January 2002), has a teacher or any other person ever talked in class about the dangers of smoking?

a. Yes
b. No
c. Not sure

53. During this school year (since January 2002), was there any discussion in any of your classesabout the reasons why people of your age smoke?

a. We did not have lessons on smoking
b. Yes, we had lessons on smoking and were told on reasons why young people smoke
c. We had lessons on smoking but were not told on reasons why young people smoke
d. Not sure

54. During this school year, was there any discussion in any of your classes about the effects of smoking, like it makes your teeth yellow, it causes wrinkles, or it makes you smell bad?

a. Yes
b. No
c. Not sure

55. How long was there any discussion on smoking and health as part of a lesson?

a. Never
b. This term
c. Last term
d. 2 terms ago
e. 3 terms ago
f. More than a year ago

THE LAST 3 QUESTIONS ASK FOR SOME BACKGROUND INFORMATION ABOUT YOURSELF.

56. How old are you? (Example: pretend you are 14 years and your birthday is in 2 weeks, then you should answer d (14 years old. You become 15 years only from the day of your 15th birthday).

a. 11 years old or younger
b. 12 years old
c. 13 years old
d. 14 years old
e. 15 years old
f. 16 years old
g. 17 years old or older

57. What is your sex?

a. Boy
b. Girl

58. In what grade are you?

a. S1
b. S2
c. S3
d. S4

59. Do you have a 'boyfriend' or 'girlfriend' currently (for at least 1 month)?

b. Yes
c. No

60. Do your 'boyfriend' or 'girlfriend' smokes?

a. Never
b. Occasionally but not every week
c. Every week

Every day

THE NEXT 6 QUESTIONS ASK ABOUT YOUR USE OF ALCOHOL.

61. At present, how often do you drink beer?

a. Never
b. Less than once a month
c. Every month, but not every week
d. Every week, but not every day
e. Every day

62. At present, how often do you drink wine?

a. Never
b. Less than once a month
c. Every month, but not every week
d. Every week, but not every day
e. Every day

63. At present, how often do you drink whiskey or liquor?

a. Never
b. Less than once a month
c. Every month, but not every week
d. Every week, but not every day
e. Every day

64. At present, how often do you drink baka, kalu or lapire?

a. Never
b. Less than once a month
c. Every month, but not every week
d. Every week, but not every day
e. Every day

65. During the past 30 days (one month), on how many days did you drink alcohol?

a. 0 days
b. 1 or 2 days
c. 3 to 5 days
d. 6 to 9 days
e. 10 to 19 days
f. 20 to 29 days
g. All 30 days

66. Have you ever had so much alcohol that you were drunk?

a No, never
b Yes, once
c Yes, 2-3 times
d Yes, 4-10 times
e. Yes, more than 10 times

THE NEXT 6 QUESTIONS ASK ABOUT YOUR USE OF DRUGS.

67. Have you ever taken joint, marijuana, or hashish in your life?

a. Never
b. Once
c. 2-4 times
d. 5 or more times
e. I don't know the meaning of 'joint, marijuana, or hashish'

68. Have you ever taken ecstasy in your life?

a. Never
b. Once
c. 2-4 times
d. 5 or more times
e. I don't know the meaning of 'ecstasy'

69. Have you ever taken cocaine in your life?

a. Never
b. Once
c. 2-4 times
d. 5 or more times
e. I don't know the meaning of 'cocaine'

70. Have you ever sniffed glue or solvent in your life?

a. Never
b. Once
c. 2-4 times
d. 5 or more times
e. I don't know the meaning of 'sniffing glue or solvent'

71. During the past 12 months, how many times did you take a joint, marijuana, or hashish?

a. I did not take a joint, marijuana, or hashish during the past year
b. One time
c. 2-3 times
d. 4-9 times
e. 10 times or more often
f. I don't know the meaning of 'joint, marijuana, or hashish'

Now all you have to do is to put the questionnaire in the box with the answer sheets
We'd like to thank you very much for your collaboration.
